# Increased risk of antiphospholipid syndrome in patients with psoriasis: a retrospective cohort study

**DOI:** 10.3389/fimmu.2025.1620768

**Published:** 2025-09-09

**Authors:** Qian Wang, Ran Cui, Yu Du, Yu-Hsun Wang, James Cheng-Chung Wei, Sheng-Ming Dai

**Affiliations:** ^1^ Department of Rheumatology and Immunology, Shanghai Sixth People’s Hospital Affiliated to Shanghai Jiao Tong University School of Medicine, Shanghai, China; ^2^ Department of Medical Research, Chung Shan Medical University Hospital, Taichung, Taiwan; ^3^ Institute of Medicine, College of Medicine, Chung Shan Medical University, Taichung, Taiwan; ^4^ Department of Allergy, Immunology and Rheumatology, Chung Shan Medical University Hospital, Taichung, Taiwan; ^5^ Graduate Institute of Integrated Medicine, China Medical University, Taichung, Taiwan; ^6^ Department of Nursing, Chung Shan Medical University, Taichung, Taiwan

**Keywords:** psoriasis, arthritis, psoriatic, retrospective study, antiphospholipid syndrome, risk factors

## Abstract

**Objectives:**

Psoriasis is associated with systemic inflammation and immune dysregulation, raising concerns about its potential link to antiphospholipid syndrome (APS). However, evidence on the risk of APS in psoriasis patients remains limited.

**Materials and methods:**

This retrospective cohort study utilized data from the TriNetX US Collaborative Networks (2002–2022). Propensity score matching (1:1) was performed to balance demographic variables, comorbidities, and medication use between 288,678 psoriasis and non-psoriasis patients. The univariate Cox proportional hazard model and subgroup analyses were used to estimate the hazard ratio for APS. The Kaplan-Meier method was used to plot the cumulative incidence curves.

**Results:**

After matching, each cohort included 288,678 patients. Psoriasis patients exhibited significantly higher APS incidence (1,349 cases vs. 673 in controls; HR: 1.71, 95% CI: 1.56–1.88). Psoriatic arthritis (PsA) further amplified risk (HR: 1.91, 95% CI: 1.58–2.31). Subgroup analyses identified elevated APS susceptibility in older adults, females, Black/African American individuals, and those with chronic comorbidities.

**Conclusions:**

Psoriasis and psoriatic arthritis are significant risk factors for APS, highlighting the need for targeted screening and management strategies in these populations.

## Introduction

Psoriasis is an inflammatory skin disease mediated by cells and molecules of both the innate and adaptive immune systems (1). The pathogenesis of psoriasis is complex, including immune response, genetic susceptibility and environmental factors. Psoriasis is a disease more than skin, and it is associated with systemic disorders (2). Patients with psoriasis are at an increased risk of developing other chronic diseases. These comorbid diseases of psoriasis include psoriatic arthritis, Crohn’s disease, cancer, depression, metabolic syndrome, and cardiovascular disorders (3, 4), imposing an excessive burden on both individuals and society. Studies have shown a connection between psoriasis and a number of autoimmune disorders, including systemic lupus erythematosus (SLE) and rheumatoid arthritis (RA) (5), indicating that these disorders share pathophysiological pathways.

Antiphospholipid syndrome (APS), characterized by thromboembolic events and persistent antiphospholipid antibodies (aPL), is strongly linked to autoimmune diseases like SLE and RA (6). Despite overlapping immunological dysfunction (e.g., autoantibody production, endothelial injury), the association between psoriasis and APS remains underexplored, necessitating rigorous epidemiological studies to clarify potential risks.

In order to reduce the influence of confounding variables, a statistical technique called propensity score matching (PSM) pairs patients with specific medical conditions with those who do not. In this study, we utilized the TriNetX database, which provides real-time electronic medical record data from a global collaborative health research network. We used PSM to adjust for demographic variables, comorbidities, and medication use, trying to investigate whether patients with psoriasis have an increased risk of developing APS. Our findings may bring new viewpoints for clinical practice and more individualized advice for psoriasis patients.

## Materials and methods

### Data sources

The sources of the data were TriNetX (https://trinetx.com), a large federated health research network that provides real-time updates of data from electronic health records, including vital statuses, laboratory results, diagnoses, treatments, and demographics. Protected health information and personal data are not made available to TriNetX platform users through the use of aggregated counts and statistical summaries of deidentified information.

The Health Insurance Portability and Accountability Act and the General Data Protection Regulation are both complied with by the TriNetX platform. As this platform only aggregated counts and statistical summaries of deidentified information, the Western Institutional Review Board waived informed consent requirements for TriNetX. In addition, the use of TriNetX for the present study was approved under the authority of the Institutional Review Board of Chung Shan Medical University Hospital (No: CS2-21176).

### Study design and patient selections

The International Classification of Diseases, Tenth Revision, Clinical Modification (ICD-10-CM) codes were used to classify diseases. The exposure cohort consisted of all patients diagnosed with psoriasis (ICD-10-CM=L40) between January 1, 2002, and December 31, 2022. Patients without psoriasis (ICD-10-CM=Z00) were thus categorized as the control group. In the two cohorts, patients diagnosed with APS (ICD10-CM=D68.61), were defined as the main outcome in this study. Patients diagnosed with APS prior to the index date were excluded from the analysis. The comorbidities analyzed in this study were hypertensive disease (ICD-10-CM=I10-I16), ischemic heart disease (ICD-10-CM=I20-I25), cerebrovascular diseases (ICD-10-CM=I60-I69), diabetes mellitus (ICD-10-CM=E08-E13), disorders of lipoprotein metabolism (ICD-10-CM=E78), liver disease (ICD-10-CM=K70-K77), and depressive disease (ICD-10-CM=F32). The comorbidities were defined as those receiving a diagnosis within one year before the index date. The other covariates, including age, sex, smoking (ICD-10-CM=Z87.891), body mass index (BMI) (Lab: TXN9084), medical utilization (outpatient visit, emergency visit, hospitalization), and medication use (hormones/synthetics/modifiers, Medication=HS000; anti-rheumatics, Medication=MS100), were also analyzed. Compared with psoriasis without PsA, the impact of PsA (ICD-10-CM=L40.5) on the incidence of APS was also examined.

### Statistical analysis

All statistical analyses were conducted using the TriNetX analytics platform. To reduce the effect of confounding factors, we applied the built-in capability of TriNetX to generate the propensity score, and to perform at a 1:1 ratio matching by using greedy nearest neighbor matching with a caliper of 0.1 pooled standard deviations of the two cohorts for demographic variables, comorbidities, and medication. Comparisons between the two cohorts before and after matching were explored with a standardized mean difference (SMD). It was considered well matched if the SMD was lower than 0.1. The Kaplan-Meier curve was used to estimate the risk of developing APS. The hazard ratio (HR) and 95% confidence interval (CI) were calculated using univariate Cox proportional hazard model. The log-rank tests were done within TriNetX to assess whether the survival curves were different between the two cohorts. A *p* value less than 0.05 was considered statistically significant. Subgroup analyses were further performed to explore whether the risk of developing APS in psoriasis patients differed by each covariate. For every subgroup analysis, propensity score matching (PSM) was carried out once more in accordance with the TriNetX network’s architecture.

## Results

### Patient characteristics

**Figure 1:**
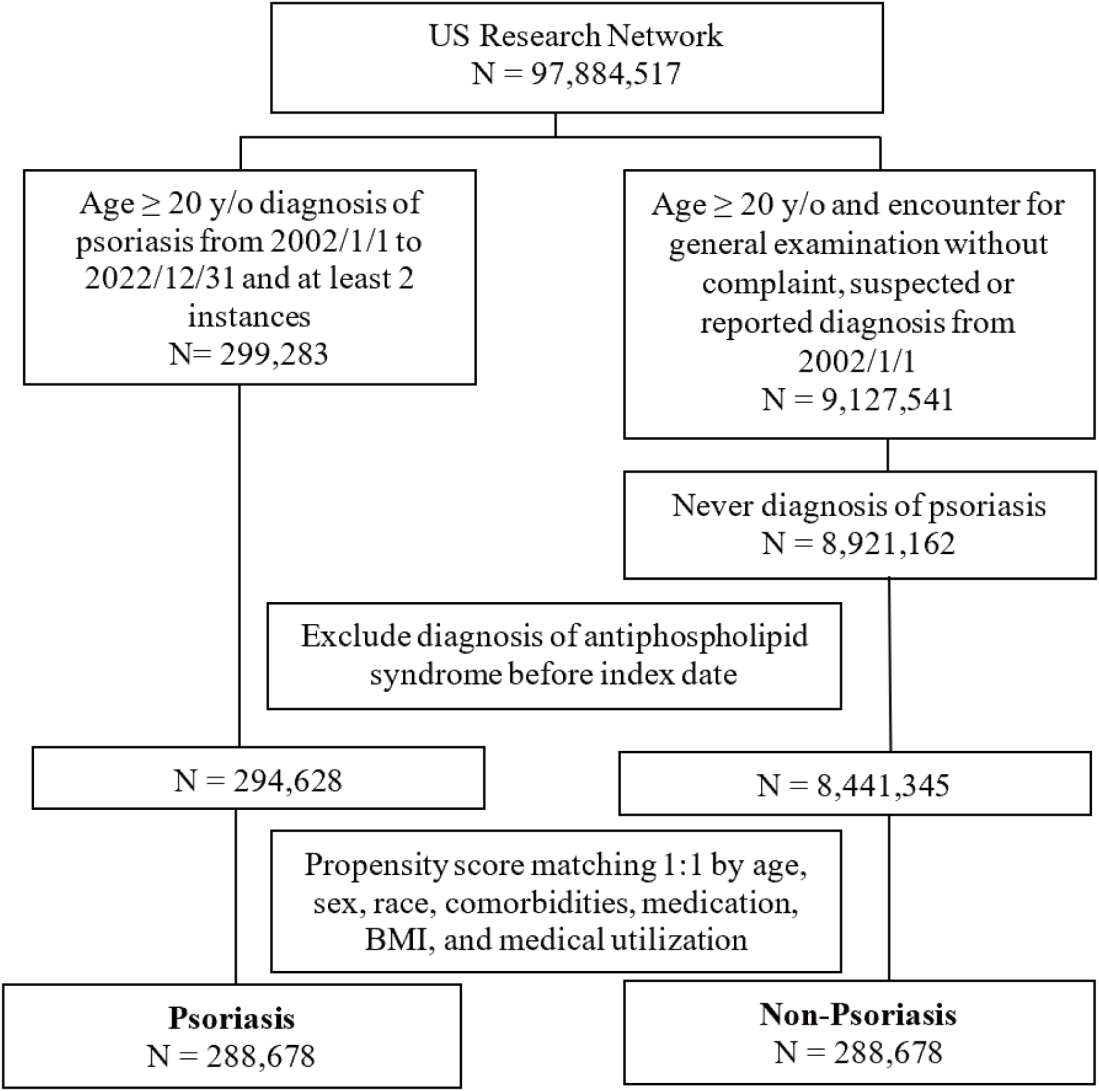
Flow chart of the study design. BMI, body mass index.

We identified 299,283 patients with psoriasis (age ≥ 20 years old) between January 1, 2002, and December 31, 2022, in the exposure cohort, and 8,921,162 non-psoriasis patients (age ≥ 20 years old) in the control cohort. After removing the APS cases diagnosed prior to the index date and performing PSM, the psoriasis and non-psoriasis cohorts of these patients ultimately comprised 288,678 patients each (**Figure 1**). The baseline characteristics, including demographic profiles, comorbidities, and medication usage of the patients before and after PSM, are listed in **Table 1**.

**Table 1:** Demographic characteristics of psoriasis cohort and non-psoriasis cohort.

	Before PSM		p	SMD	After PSM		p	SMD
	Psoriasis N = 294,628	Non-psoriasis N = 8,441,345			Psoriasis N = 288,678	Non-psoriasis N = 288,678		
Age (yrs), mean ± SD	51.8 ± 15.7	47.8 ± 17.1	<0.001	0.239	51.8 ± 15.7	51.8 ± 15.7	0.996	<0.001
Sex, n (%)								
Female	160857 (55.7)	4764701 (56.9)	<0.001	0.023	160857 (55.7)	160871 (55.7)	0.970	<0.001
Male	127345 (44.1)	3530122 (42.1)	<0.001	0.040	127345 (44.1)	126124 (43.7)	0.001	0.009
Race, n (%)								
White	228929 (79.3)	5416810 (64.6)	<0.001	0.331	228929 (79.3)	228935 (79.3)	0.984	<0.001
Black or African American	16639 (5.8)	1276495 (15.2)	<0.001	0.313	16639 (5.8)	16633 (5.8)	0.973	<0.001
Asian	7012 (2.4)	276035 (3.3)	<0.001	0.052	7012 (2.4)	7514 (2.6)	<0.001	0.011
Comorbidities, n (%)								
Hypertensive disease	58161 (20.1)	1407184 (16.8)	<0.001	0.087	58161 (20.1)	58147 (20.1)	0.963	<0.001
Ischemic heart disease	16017 (5.5)	313313 (3.7)	<0.001	0.086	16017 (5.5)	14282 (4.9)	<0.001	0.027
Cerebrovascular diseases	9457 (3.3)	164860 (2.0)	<0.001	0.082	9457 (3.3)	7493 (2.6)	<0.001	0.040
Diabetes mellitus	30586 (10.6)	619868 (7.4)	<0.001	0.112	30586 (10.6)	30573 (10.6)	0.956	<0.001
Disorders of lipoprotein metabolism	47169 (16.3)	1131776 (13.5)	<0.001	0.080	47169 (16.3)	47163 (16.3)	0.983	<0.001
Liver disease	11342 (3.9)	152307 (1.8)	<0.001	0.127	11342 (3.9)	7847 (2.7)	<0.001	0.068
Depressive disease	21576 (7.5)	407629 (4.9)	<0.001	0.109	21576 (7.5)	18946 (6.6)	<0.001	0.036
Smoking	16097 (5.6)	238112 (2.8)	<0.001	0.137	16097 (5.6)	12274 (4.3)	<0.001	0.061
Medication, n (%)								
Hormones/synthetics/modifiers	96315 (33.4)	1826589 (21.8)	<0.001	0.261	96315 (33.4)	96323 (33.4)	0.982	<0.001
Antirheumatics	53872 (18.7)	937475 (11.2)	<0.001	0.211	53872 (18.7)	53848 (18.7)	0.935	<0.001
BMI (kg/m^2^), mean ± SD	30.2 ± 6.9	29.1 ± 6.7	<0.001	0.158	30.2 ± 6.9	29.4 ± 6.7	<0.001	0.120
BMI (kg/m^2^), n (%)								
<18.5	1479 (0.5)	42848 (0.5)	0.942	<0.001	1479 (0.5)	1873 (0.6)	<0.001	0.018
18.5-24.9	15304 (5.3)	397236 (4.7)	<0.001	0.026	15304 (5.3)	16852 (5.8)	<0.001	0.023
25-29.9	21633 (7.5)	474041 (5.7)	<0.001	0.074	21633 (7.5)	21428 (7.4)	0.304	0.003
≥30	29759 (10.3)	549136 (6.6)	<0.001	0.135	29759 (10.3)	25501 (8.8)	<0.001	0.050
Medical utilization, n (%)								
Outpatient	177717 (61.6)	4398343 (52.5)	<0.001	0.184	177717 (61.6)	177726 (61.6)	0.981	<0.001
Emergency	26743 (9.3)	739646 (8.8)	<0.001	0.015	26743 (9.3)	30298 (10.5)	<0.001	0.041
Hospitalization	29981 (10.4)	659635 (7.9)	<0.001	0.087	29981 (10.4)	30849 (10.7)	<0.001	0.010

SMD, standardized mean difference; PSM, propensity score matching; SD, standard deviation; BMI, body mass index.

Before PSM, patients with psoriasis were older than those without, with mean age 51.8 ± 15.7 years and 47.8 ± 17.1 years, respectively. The psoriasis cohort comprised a substantially larger percentage of White people and lower percentages of Black or African American and Asian people, compared to the control cohort. After PSM, the demographics, comorbidities, and medication use of the two groups were similar (all SMDs <0.1).

### Risk of APS in psoriasis and non-psoriasis cohorts and subgroup analyses

**Figure 2:**
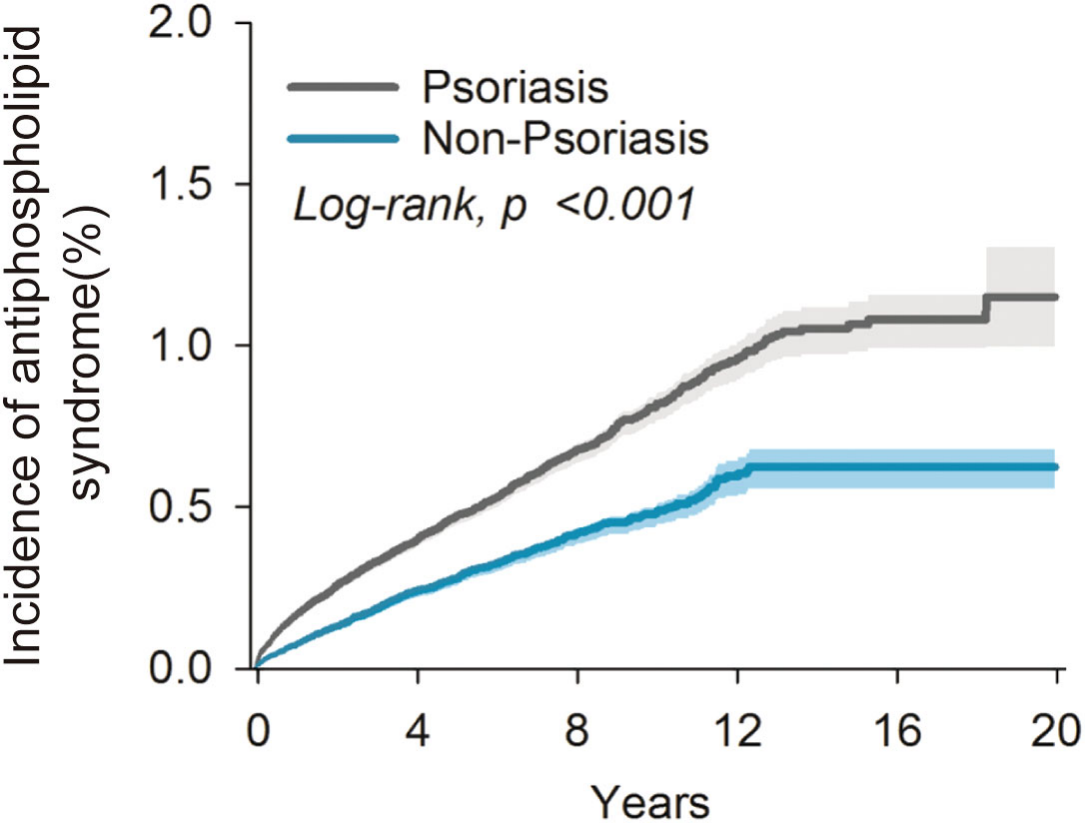
Kaplan-Meier analysis for risk of antiphospholipid syndrome.

In the psoriasis cohort, there were 1,349 incident cases of APS during the study period. In the non-psoriasis cohort, there were 673 incident cases of APS. The risk of developing APS in the psoriasis cohort was significantly higher than in the non-psoriasis cohort with a HR of 1.71(95% CI 1.56 to 1.88). Otherwise, the risk of developing APS in the PsA cohort was significantly higher than in the non-psoriasis cohort with a HR of 1.91 (95%CI 1.58 to 2.31). Patients with PsA have an even higher risk of APS than those with psoriasis alone (**Table 2**). The Kaplan-Meier curves revealed that the incidence of APS in the psoriasis cohort was significantly higher than in the non-psoriasis cohort (log-rank test, p<0.001) (**Figure 2**). We conducted an additional sensitivity analysis including patients with psoriasis who received methotrexate or TNF inhibitors. The results showed that psoriasis with disease modifying anti-rheumatic drugs was significantly associated with antiphospholipid syndrome (HR = 1.58, 95% CI: 1.34–1.87) (**Supplementary Table 1**). These findings indicated that psoriasis may increase the risk of developing APS.

**Table 2:** Risk of antiphospholipid syndrome exposed to psoriasis compared to non-psoriasis.

	Before PSM			After PSM		
	N	No. of event	HR (95% C.I.)	N	No. of event	HR (95% C.I.)
Group						
Non-Psoriasis	8,425,385	1,352	Reference	288,678	673	Reference
Psoriasis	292,106	1,352	1.88 (1.78–1.99)	288,678	1,349	1.71 (1.56–1.88)
Group						
Non-Psoriasis	8,079,123	16,110	Reference	64,912	157	Reference
Psoriatic arthritis	69,227	348	2.13 (1.91–2.37)	64,912	340	1.91 (1.58–2.31)

PSM, propensity score matching; HR, hazard ratio; CI, confidence interval.

**Figure 3:**
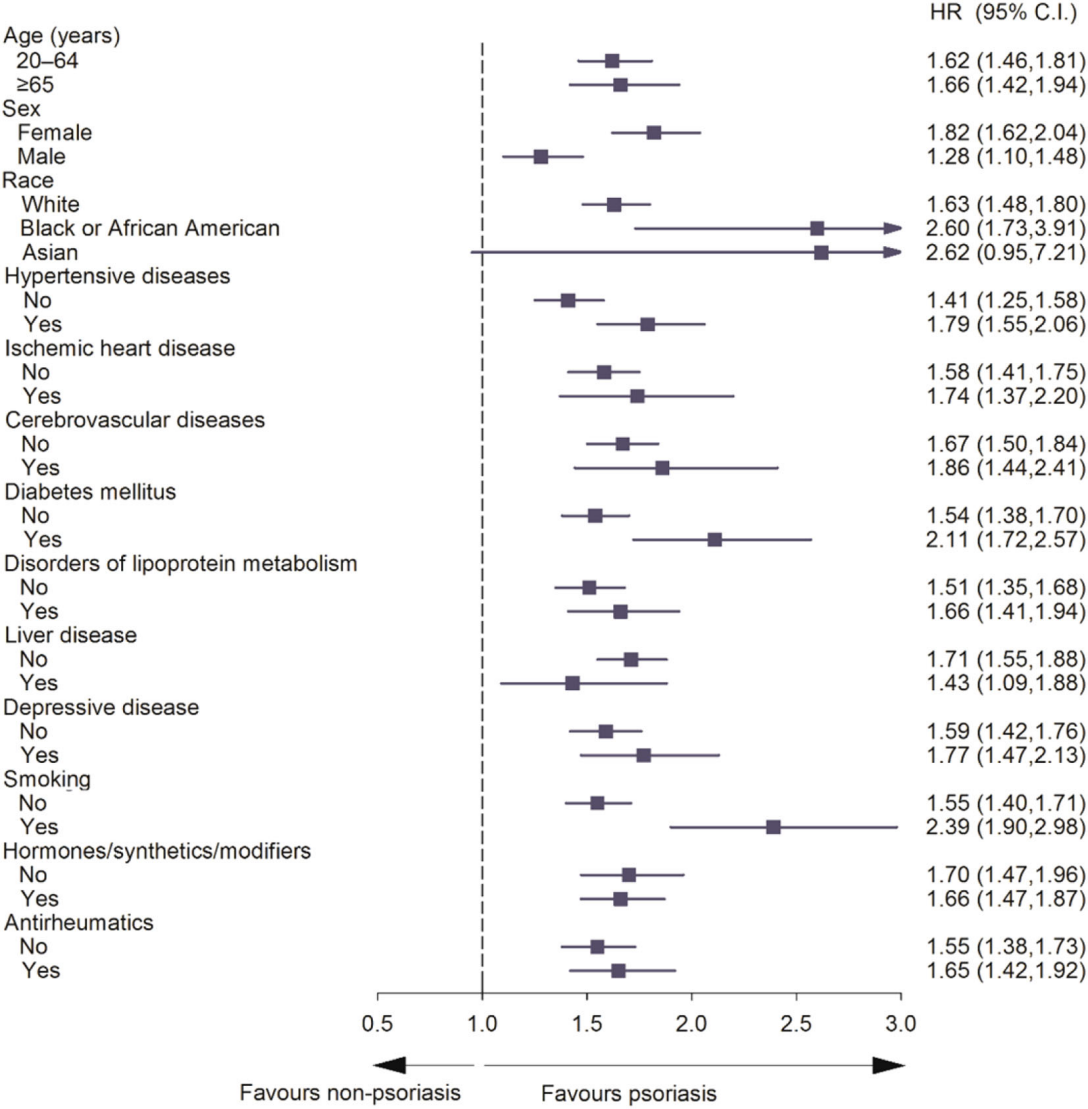
Forest plot for subgroup analysis of risk of antiphospholipid syndrome exposed to psoriasis compared to non-psoriasis.

We then classified the patients into different subgroups based on age, sex, race and comorbidities. The subgroup analysis also confirmed the higher risks of APS in patients with psoriasis (**Figure 3**). In these subgroups, psoriasis was associated with a higher risk of developing APS in ≥65 aged populations (HR: 1.66, 95% CI: 1.42–1.94), females (HR: 1.82, 95% CI: 1.62–2.04), Black or African American individuals (HR: 2.60, 95% CI: 1.73–3.91), individuals with hypertensive diseases (HR: 1.79, 95% CI: 1.55–2.06), ischemic heart diseases (HR: 1.74, 95% CI: 1.37–2.20), cerebrovascular diseases (HR: 1.86, 95% CI: 1.44–2.41), diabetes mellitus (HR: 2.11, 95% CI: 1.72–2.57), disorders of lipoprotein metabolism (HR: 1.66, 95% CI: 1.41–1.94), liver disease (HR: 1.43, 95% CI: 1.09–1.88), depressive disease (HR:1.77, 95%CI: 1.47–2.13) and smoking (HR:2.39, 95%CI: 1.90–2.98). These results implied that psoriasis may make some particular groups more susceptible to APS.

## Discussion

In this study, we investigated the impact of psoriasis on the risk of APS using a large-scale, real-world data from the TrinetX network. We compared with the non-psoriasis group, patients with psoriasis or patients with PsA are more likely to develop APS. The risk of APS in psoriasis group was higher in non-psoriasis group after conducting PSM and adjusting for potential confounders. The subgroups also indicated higher risks of developing APS in patients with psoriasis. To our knowledge, this is the first study to investigate the risk of APS in patients with psoriatic diseases.

A previous study found that patients with psoriatic arthritis have a higher odds ratio for the development of SLE, Sjögren syndrome, systemic sclerosis, celiac disease, giant cell arteritis, hemolytic anemia and even surgery (7, 8). Our study demonstrated an association between psoriatic disease and APS, consequently providing novel viewpoints on the risk variables that underlie this correlation.

Here are some possible explanations for this connection. Psoriasis is an inflammatory mediated disease. The pathophysiology of the development of psoriasis involves both the innate and adaptive immune systems (9). There is a brisk immune cell infiltrate composed of dendritic cells (DCs) and CD4^+^ T cells within the upper papillary dermis and CD8^+^ T cells within the epidermis (10). CD4^+^ and CD8^+^ T cells producing IL-17 have been identified in psoriasis lesions (11, 12). Additionally, there was an increase in the production of interferon γ (IFN-γ), tumor necrosis factor α (TNF-α), interleukin 1β (IL-1β), IL-22, and IL-23 (13). While in APS, immunothrombosis participated in the pathogenesis. Anti-beta-2 glycoprotein I antibodies (a major pathogenic antibody in APS) engage TLR4 signaling and trigger monocytes to express tissue factors and proinflammatory cytokines such as TNF-α and IL-1β (14). The subsequent activation of the innate immune response was considered the second hit in the thrombotic mechanism in APS (15). Besides, high levels of neutrophil extracellular traps (NETs) remnants are found in the circulation of patients with APS (16). NETs can trigger thrombosis in the original place through multiple mechanisms in APS (17). While, in psoriasis, extracellular DNA has recently been shown in the epidermis in association with NETs (18).

On the other hand, the role of complement activation is confirmed in APS. Complement products C3a and C5a were believed to cause placental inflammation (19). Activation of the complement cascade might provoke thrombosis (20). There is evidence supporting activation of the classical and alternative complement pathways in patients with catastrophic APS (21), and eculizumab (a humanized anti-C5a monoclonal antibody) has been successfully used in catastrophic APS (22). However, C3a and C5a were found in psoriasis lesions for nearly four decades (23). The complement activation may contribute to the relationship between psoriasis and APS. There is also some common mechanism between psoriasis and APS, such as endothelial cell activation and the production of vascular endothelial growth factor (VEGF) (24, 25).

Our subgroup studies shed additional light on which psoriasis individuals might be most susceptible to APS. Sex hormones and genetic predispositions may be the cause of the well-documented female tendency for autoimmune diseases like APS, there is a subtype of APS named obstetric APS (26). A number of factors, such as sample size, genetic differences, socioeconomic inequities, and access to healthcare, are probably involved in the relatively high risk of APS among Black or African American individuals with psoriasis. Asians did not have a higher risk, which might be because they have comparatively lower disease activities. Other chronic diseases like diabetes mellitus, metabolic disorders and ischemic heart disease were more common in APS (27, 28). Our results highlight the need for personalized screening and management strategies for patients with psoriasis at the greatest risk for comorbid conditions.

However, there are several potential limitations in the present study. First, the follow-up data of patients is not available in the TriNetX platform. So, we cannot provide the follow-up time of the study individuals. Second, the data were sourced from TriNetX, which was developed to collect information primarily from US and European health care organizations, and therefore may not be representative of the broader world population. Third, confounding bias may exist in this retrospective study even through we used propensity score matching to balance the covariates, and we adjusted for baseline characteristics. Additionally, this study was unable to capture the specific clinical manifestations regarding APS phenotypes (e.g., thrombotic, obstetric, non-thrombotic, or combined presentations) or antibody profiles (anticardiolipin, anti-β2 glycoprotein I, and lupus anticoagulant) used to confirm the diagnosis of antiphospholipid syndrome (APS), as such information is not available in the database. The identification of APS relied on ICD-10-CM coding assigned by physicians, diagnostic codes may not distinguish between clinical diagnosis and formal classification, which can lead to over- or underestimation of APS cases.

In conclusion, we conducted a large-scale retrospective cohort study to investigate the impact of psoriasis on the incidence of APS. We revealed that there is an increased risk of APS in patients with psoriasis and patients with psoriatic arthritis. Psoriatic diseases have a more intensive relationship with connective tissue diseases.

## Data Availability

The original contributions presented in the study are included in the article/**Supplementary Material**. Further inquiries can be directed to the corresponding authors.
