# Supplementary material for: Increased risk of antiphospholipid syndrome in patients with psoriasis: a retrospective cohort study
Source: Front Immunol. 2025 Sep 9;16:1620768. doi: 10.3389/fimmu.2025.1620768 (PMC12454023; doi:10.3389/fimmu.2025.1620768)
Supplement: Supplementary file 1 [file Table1.docx]

**Supplementary Table 1** Sensitivity analysis for risk of antiphospholipid syndrome

|  | HR (95% C.I.) |
| --- | --- |
| Group |  |
| Non-Psoriasis | Reference |
| Psoriasis with DMARDs* | 1.58 (1.34–1.87) |
| Group |  |
| Non-Psoriasis | Reference |
| Psoriasis with corticosteroids | 1.63 (1.36–1.95) |
| Venous thromboembolism/pulmonary embolism |  |
| Non-Psoriasis | Reference |
| Psoriasis | 1.35 (0.98–1.87) |

*Psoriasis treated with methotrexate (RxNorm: 6851) or TNF inhibitor (TNFi) therapy, including etanercept (RxNorm: 214555), adalimumab (RxNorm: 327361), infliximab (RxNorm: 191831), and certolizumab pegol (RxNorm: 709271). Corticosteroids (ATC: H02AB). RxNorm: Medical prescription normalized Medical prescription. ATC: Anatomical Therapeutic Chemical.

Venous thromboembolism: ICD-10-CM = I82.4; Pulmonary embolism: ICD-10-CM = I26.

DMARDs: disease modifying anti-rheumatic drugs.
